# Preoperative hidden renal dysfunction add an age dependent risk of progressive chronic kidney disease after cardiac surgery

**DOI:** 10.1186/s13019-019-0977-9

**Published:** 2019-08-22

**Authors:** Jiarui Xu, Jiawei Yu, Xialian Xu, Bo Shen, Yimei Wang, Wuhua Jiang, Wenlv Lv, Yi Fang, Zhe Luo, Chunsheng Wang, Jie Teng, Xiaoqiang Ding

**Affiliations:** 1 Department of Nephrology, Zhongshan Hospital, Shanghai Medical College, Fudan University, No. 180 Fenglin Road, Shanghai, 200032 China; 2 Shanghai Medical Center of Kidney, No. 180 Fenglin Road, Shanghai, 200032 China; 3 Shanghai Institute for Kidney and Dialysis, No. 180 Fenglin Road, Shanghai, 200032 China; 4 Shanghai Key Laboratory of Kidney and Blood Purification, No. 180 Fenglin Road, Shanghai, 200032 China; 5 Hemodialysis Quality of Control Center of Shanghai, No. 180 Fenglin Road, Shanghai, 200032 China; 6 Department of Critical Care Medicine, Zhongshan Hospital, Shanghai Medical College, Fudan University, No. 180 Fenglin Road, Shanghai, 200032 China; 7 Department of Cardiovascular Surgery, Zhongshan Hospital, Shanghai Medical College, Fudan University, No. 180 Fenglin Road, Shanghai, 200032 China; 8 Department of Nephrology, Xiamen Branch, Zhongshan Hospital, Fudan University, No. 668 Jinhu Road, Xiamen, 361015 Fujian China

**Keywords:** Cardiac surgery, Estimated glomerular filtration rate, Acute kidney injury, Progressive chronic kidney disease

## Abstract

**Background:**

To study different value of estimated glomerular filtration rate with normal serum creatinine whether is a risk factor for hidden renal function of cardiac surgery outcomes.

**Methods:**

A total of 1744 cardiac surgery patients with serum creatinine ≤1.2 mg/dL (female)/1.5 mg/dL (male) were divided into 3 groups: estimated glomerular filtration rate ≥ 90 mL/min/1.73 m^2^ (no renal dysfunction, *n* = 829), 60 ≤ estimated glomerular filtration rate < 90 mL/min/1.73 m^2^ (hidden renal dysfunction, *n* = 857), estimated glomerular filtration rate < 60 mL/min/1.73 m^2^ (known renal dysfunction, *n* = 58) and followed up for 3 years. Multivariate regression analyses for risk factors of postoperative acute kidney injury.

**Results:**

The proportion of preoperative hidden renal dysfunction was 67.1% among patients ≥  65 years old and 44.1% among patients < 65 years old. Multivariate Cox regression analyses showed that for patients < 65 years, known renal dysfunction was a risk factor for postoperative acute kidney injury (*P* <  0.01) and progressive chronic kidney disease (*P* = 0.018), while hidden renal dysfunction was a risk factor for progressive chronic kidney disease (*P* = 0.024). For patients ≥  65 years, only known renal dysfunction was a risk factors for 3-year mortality (*P* = 0.022) and progressive chronic kidney disease (*P* <  0.01).

**Conclusion:**

Hidden renal dysfunction was common in patients with normal serum creatinine for cardiac surgery, with a prevalence of 49.1%. For patients < 65 years old, hidden renal dysfunction was an independent risk factor for progressive chronic kidney disease.

## Background

Preoperative renal dysfunction is a high risk factor for outcomes in cardiac surgery, not only for postoperative acute kidney injury (AKI), but also for in-hospital mortality and even long-term outcomes [1–3]. Renal dysfunction is mostly evaluated by serum creatinine (SCr), but scholars have noted that patients with normal SCr had estimated glomerular filtration rate (eGFR) values < 60 mL/min and called it “occult renal dysfunction”, which significantly increased the risk of renal replacement therapy (RRT), inpatient death and prolonged hospitalization after coronary artery bypass grafting (CABG) surgery [4, 5]. In the study of Volkmann et al., patients with plasma creatinine (PCr) ≤ 1.5 mg/dL who underwent CABG were divided into 2 groups, estimated creatinine clearance (eCrCl) ≥ 60 mL/min (normal renal function) or eCrCl < 60 mL/min (reduced renal function), respectively. The results showed that reduced renal function had a double risk of death, a longer total hospital stay and post-surgical hospital stay than those patients with normal renal function [6]. However, a considerable fraction of cardiac surgery patients may have normal SCr values and an eGFR under 90 mL/min/1.73 m^2^, but above 60 mL/min/1.73 m^2^, particularly since eGFR declines with age [7]. In the present retrospective, observational, single center study we analyzed whether patients with “normal” SCr and “hidden renal dysfunction” (60 ≤ eGFR < 90 mL/min) have an enhanced risk of complications after cardiac surgery especially focusing on age-related eGFR decline.

## Methods

### Patients

In this retrospective, observational study, we collected data from patients who underwent cardiac surgery and with preoperative SCr ≤ 1.2 mg/dL (female) / 1.5 mg/dL (male) in Shanghai Zhongshan Hospital between October 2012 and July 2013. Exclusion criteria were < 18 years old; preoperative SCr ≥  1.2 mg/dL (female) / 1.5 mg/dL (male) and received deep hypothermic circulatory arrest or heart transplantation. The Ethical Committee of Zhongshan Hospital affiliated to Fudan University approved the study (No. B2017–039) and written informed consent was obtained from all patients and our study was performed in accordance with the Declaration of Helsinki regarding the ethical principles for medical research involving human subjects.

### Definitions and groups

Pre-operative hidden renal dysfunction was defined as SCr ≤ 1.2 mg/dL (female)/1.5 mg/dL (male) and 60 ≤ eGFR < 90 mL/min/1.73 m^2^. AKI was defined according to the KDIGO 2012 criteria as the absolute value of the SCr increase ≥  26.5 μmol/L within 48 h or an increase > 50% compared to the baseline values within 7 days, or a urine output < 0.5 mL/kg/h ≥ 6 h [8].

Progressive chronic kidney disease (CKD) was defined as CKD stages 4–5 (eGFR ≤  30 mL/min/1.73 m^2^) including End-stage renal disease (ESRD) (receive maintenance renal replacement therapy or renal transplantation). CKD was diagnosed according to the Kidney Disease: Improving Global Outcomes (KDIGO) 2002 criteria [9]. All enrolled patients were divided into three groups: eGFR ≥  90 mL/min/1.73 m^2^ (no renal dysfunction), 60 ≤ eGFR < 90 mL/min/1.73 m^2^ (hidden renal dysfunction) and eGFR < 60 mL/min/1.73 m^2^ (known renal dysfunction). Low cardiac output syndrome (LCOS) was diagnosed when patients had two or more of the following [10, 11]: (1) decreased systolic pressure > 20% of the basic preoperative value lasting ≥  2 h; (2) signs of impairment of body perfusion (cold extremities, lowered level of consciousness, or a combination of these signs) and lasting ≥  2 h; (3) need for at least three vasoactive drugs (dopamine, dobutamine, epinephrine or norepinephrine) or required an intra-aortic balloon pump (IABP). Intra-operative hypotension was defined as mean arterial pressure <  65 mmHg and lasting ≥  10 min. High dose of vasoactive agents was defined as need of at least three vasoactive drugs, or the dose of norepinephrine or epinephrine more than 0.3 μg/kg/min. All the patients were followed up for 3 years through telephone, e-mail and hospital visits until October 2016. The primary endpoint was all causes of mortality and progressive CKD.

### Statistical analysis

Statistical analysis was conducted with SPSS Statistics for Windows (Version 22.0, SPSS Inc., Chicago, US). Normally distributed data are presented as means ± SD; groups were compared using two independent sample *t*-tests or ANOVA. Nonparametric data are expressed as medians (*P*_25_, *P*_75_). The Wilcoxon test was used to assess two dependent variables, a non-parametric Mann–Whitney test for independent variables, and a chi-squared test for group comparisons. Multivariate Logistic regression analysis was used to investigate the influence of multiple factors of AKI incidence. Multivariate Cox regression analysis was used to investigate the effects of multiple factors on 3-year mortality and progressive CKD. A *P*-value < 0.05 was considered to be statistically significant.

## Results

### Baseline characteristics

**Fig. 1:**
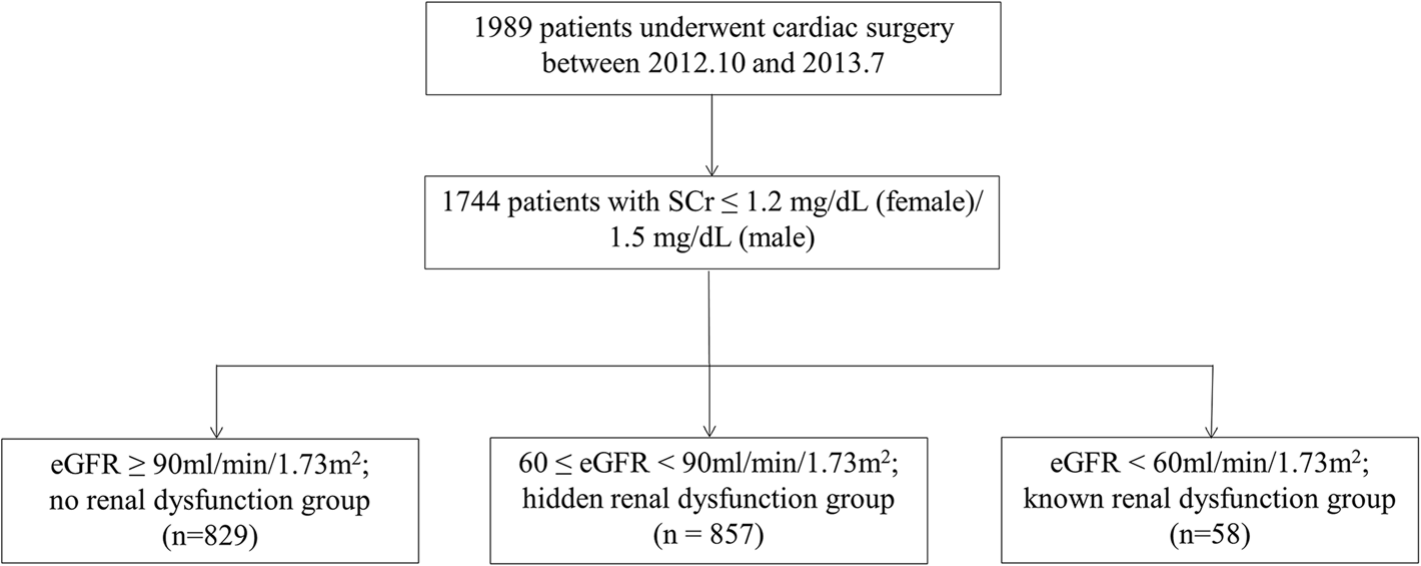
Groupings of patients. Abbreviations: SCr, serum creatinine; eGFR, estimated glomerular filtration rate

A total of 1744 patients were enrolled between October 2012 and July 2013. There were 47.53% (*n* = 829) in the eGFR ≥  90 mL/min/1.73 m^2^ group (no renal dysfunction), 49.14% (*n* = 857) in the 60 ≤ eGFR < 90 mL/min/1.73 m^2^ group (hidden renal dysfunction) and 3.33% (*n* = 58) in the eGFR < 60 mL/min/1.73 m^2^ group (known renal dysfunction) (Fig. 1).

Ages in hidden renal dysfunction group was significantly higher than in the no renal dysfunction group. The preoperative blood urea nitrogen (BUN) and SCr and uric acid in the hidden renal dysfunction group was significantly higher than in the no renal dysfunction group, but significantly lower than in the known renal dysfunction group. The proportion of proteinuria and renal ultrasound abnormal in hidden renal dysfunction group was significantly lower than in the known renal dysfunction group. The incidence of intra-operative hypotension in hidden renal dysfunction group was significantly lower than in the known renal dysfunction group (Table 1).

**Table 1:** Baseline characteristics of patients with different degree of hidden renal dysfunction

	Total (*n* = 1744)	eGFR ≥ 90 (no renal dysfunction) (*n* = 829)	60 ≤ eGFR < 90 (hidden renal dysfunction) (*n* = 857)	eGFR < 60 (known renal dysfunction) (*n* = 58)
Pre-operation				
Age	53.00 ± 14.00	48 ± 15	58 ± 12 ^a^	61 ± 15
Gender (male)	1003 (57.51%)	485 (58.50%)	483 (56.36%)	35 (60.34%)
BMI (kg/m^2^)	22.8 ± 3.5	22.4 ± 3.6	23.1 ± 3.4 ^a^	23.5 ± 3.1
Hypertension (n, %)	540 (30.96%)	223 (26.90%)	290 (33.84%)^b^	27 (46.55%)
DM (n, %)	200 (11.47%)	89 (10.74%)	100 (11.67%)	11 (18.97%)
NYHA > II (n, %)	1055 (60.49%)	445 (53.68%)	571 (66.63%)	39 (67.24%)
LVEF (%)	63.00 ± 6.00	68.00 ± 6.00	61.00 ± 5.00 ^a^	60.00 ± 5.00
BUN (mmol/L)	6.60 ± 2.20	5.90 ± 1.60	7.00 ± 2.20 ^ab^	9.60 ± 3.40
SCr (mg/dL)	0.87 ± 0.22	0.74 ± 0.12	0.96 ± 0.15 ^ab^	1.09 ± 0.12
Uric acid (μmol/L)	366.40 ± 114.30	334.10 ± 98.80	390.20 ± 112.50 ^ab^	457.20 ± 126.70
Proteinuria (n, %)	140 (8.03%)	65 (7.84%)	63 (7.35%)^b^	12 (20.69%)
Urinalysis abnormal (n, %)	279 (16.00%)	132 (15.92%)	134 (15.64%)	13 (22.41%)
Renal ultrasound abnormal (n, %)	32 (1.83%)	9 (1.09%)	17 (1.98%) ^b^	6 (10.34%)
Hemoglobin (g/L)	133.80 ± 19.60	134.30 ± 20.40	133.50 ± 18.60	130.40 ± 21.50
Platelet (10 ^9^/L)	181.00 ± 61.20	183.90 ± 64.40	178.10 ± 57.40	181.10 ± 68.10
Intra-operation				
Types of surgery (n, %)				
− Valve	903 (51.78%)	434 (52.35%)	445 (51.93%)	24 (41.38%)
− CABG/OPCAB	309 (17.72%)	111 (13.39%)	185 (21.59%)	13 (22.41%)
− Aneurysm	108 (6.19%)	57 (6.88%)	42 (4.90%)	9 (15.52%)
− Combined	140 (8.03%)	58 (7.00%)	74 (8.63%)	8 (13.79%)
CPB duration (min)	96.10 ± 40.90	94.40 ± 39.70	96.70 ± 41.20 ^b^	112.70 ± 50.10
Aortic clamping time (min)	47.40 ± 42.30	47.90 ± 43.40	46.90 ± 41.30	47.10 ± 39.30
Intra-operative hypotension (n, %)	135 (7.7%)	58 (7.0%)	68 (7.9%) ^b^	9 (15.5%)
Post-operation				
High dose of vasoactive agents (n, %)	271 (15.5%)	104 (12.5%)	153 (17.9%)	14 (24.1%)
LCOS (n, %)	118 (6.8%)	51 (6.1%)	59 (6.9%)	8 (13.9%)

*Abbreviations*: *eGFR* estimated glomerular filtration rate, *BMI* body mass index, *DM* diabetes mellitus, *NYHA* New York Heart Association, *LVEF* left ventricular ejection fraction, *BUN* blood urea nitrogen, *SCr* serum creatinine, *CABG* coronary artery bypass grafting, *OPCAB* off-pump coronary artery bypass, *CPB* cardiopulmonary bypass, *LCOS* low cardiac output syndrome

^a^Compared with group eGFR ≥90, *P* <  0.05; ^b^Compared with group eGFR < 60, *P* <  0.05

### AKI incidence and 3-year outcomes

The AKI incidence of the overall patients was 34.29% (*n* = 598). There was no statistical difference of AKI incidence between the hidden renal dysfunction and no renal dysfunction group or known renal dysfunction group. The in-hospital mortality in the hidden renal dysfunction group was significantly higher than in the no renal dysfunction group. The 3-year mortality and incidence of progressive CKD in the hidden renal dysfunction group was significantly higher than in the no renal dysfunction group, but significantly lower than in the known renal dysfunction group (Table 2).

**Table 2:** AKI incidence and 3-year outcomes of patients with different degree of hidden renal dysfunction

		eGFR ≥ 90 (no renal dysfunction)	60 ≤ eGFR < 90 (hidden renal dysfunction)	eGFR < 60 (known renal dysfunction)
All (*n* = 1744)	N	829 (47.53%)	857 (49.14%)	58 (3.33%)
	AKI incidence	271 (32.69%)	301 (35.12%)	26 (44.83%)
	In-hospital mortality	19 (2.29%)	40 (4.67%) ^a^	3 (5.17%)
	3-year mortality	61 (7.36%)	103 (12.02%) ^ab^	18 (31.03%)
	Progressive CKD incidence	22 (2.65%)	52 (6.07%) ^ab^	9 (15.52%)
< 65 years (*n* = 1356)	N	728 (53.69%)	597 (44.03%)	31 (2.29%)
	AKI incidence	223 (30.63%)	187 (31.32%)	13 (41.94%)
	In-hospital mortality	15 (2.06%)	28 (4.69%) ^a^	1 (3.23%)
	3-year mortality	36 (4.95%)	37 (6.20%)^b^	5 (16.13%)
	Progressive CKD incidence	18 (2.47%)	32 (5.36%) ^a^	4 (12.90%)
≥ 65 years (*n* = 388)	N	101 (26.03%)	260 (67.01%)	27 (6.96%)
	AKI incidence	48 (47.52%)	114 (43.85%)	13 (48.15%)
	In-hospital mortality	4 (3.96%)	12 (4.62%)	2 (7.41%)
	3-year mortality	25 (24.75%)	66 (25.38%)^b^	13 (48.15%)
	Progressive CKD incidence	4 (3.96%)	20 (7.69%)	5 (18.52%)

*Abbreviations*: *eGFR* estimated glomerular filtration rate, *AKI* acute kidney injury, *CKD* chronic kidney disease

^a^Compared with group eGFR ≥90, *P* <  0.05; ^b^Compared with group eGFR < 60, *P* <  0.05

#### Age <  65 years

The AKI incidence was 31.19% (*n* = 423) of patients under the age of 65 years. There was no statistical difference of AKI incidence between the hidden renal dysfunction and no renal dysfunction group or known renal dysfunction group. The 3-year mortality in the hidden renal dysfunction group was significantly lower than in the known renal dysfunction group. The in-hospital mortality and incidence of progressive CKD in hidden renal dysfunction was significantly higher than in no renal dysfunction group (Table 2).

#### Age ≥ 65 years

The AKI incidence was 45.10% (*n* = 175) of patients > 65 years old. There was no statistical difference of AKI incidence between the hidden renal dysfunction and no renal dysfunction group or known renal dysfunction group. The 3-year mortality in the hidden renal dysfunction group was significantly lower than in the known renal dysfunction group. There was no statistical difference of in-hospital mortality and incidence of progressive CKD between the hidden renal dysfunction and no renal dysfunction group or known renal dysfunction group (Table 2).

### Analysis of risk factors for postoperative AKI

#### Age < 65 years

Multivariate Logistic regression analysis showed that age, gender (male), hypertension, aorta surgery, CPB time, LCOS, preoperative eGFR < 60 mL/min/1.73 m^2^ and intraoperative hypotension were independent risk factors of AKI after cardiac surgery (Table 3).

**Table 3:** Multivariate logistic regression analysis for the risk factors of postoperative AKI

	Age < 65 years			Age ≥ 65 years		
	OR	95% CI	*P* value	OR	95% CI	*P*-value
Age	1.030	1.012–1.078	< 0.01	1.108	1.017–1.230	< 0.01
Gender (male)	2.451	1.890–3.378	< 0.01	2.125	1.435–4.160	< 0.01
BMI				1.078	1.011–1.146	0.023
Hypertension	1.389	1.028–1.785	0.022			
Aorta surgery	1.809	1.088–2.847	0.013			
CPB time	1.010	1.006–1.013	< 0.01			
eGFR ≥90 mL/min/1.73 m^2^	Reference	–	–			
60 ≤ eGFR < 90 mL/min/1.73 m^2^	1.729	0.764–3.021	0.257			
eGFR < 60 mL/min/1.73 m^2^	1.326	1.078–2.897	< 0.01			
Intraoperative hypotension	3.338	1.204–6.016	< 0.01	2.013	1.034–3.145	< 0.01
LCOS	2.834	1.124–4.576	< 0.01	2.126	1.018–3.896	< 0.01

*Abbreviations*: *BMI* body mass index, *CPB* cardiopulmonary bypass, *eGFR* estimated glomerular filtration rate, *LCOS* low cardiac output syndrome

Multivariate Cox regression analysis showed that age, diabetes, postoperative AKI, length of ICU stay were independent risk factors of 3-year mortality (Table 4), and age, diabetes, 60 ≤ eGFR < 90 mL/min/1.73 m^2^, eGFR < 60 mL/min/1.73 m^2^, postoperative AKI and length of ICU stay were independent risk factors of 3-year progressive CKD (Table 5).

**Table 4:** Multivariate cox regression analysis for the risk factors of 3-year mortality

	Age < 65 years			Age ≥ 65 years		
	OR	95%CI	*P*-value	OR	95%CI	*P*-value
Age	1.030	1.001–1.052	0.044			
DM	4.090	2.476–6.751	< 0.01	3.670	2.475–5.455	< 0.01
Postoperative AKI	4.970	2.849–8.661	< 0.01			
ICU stay	1.002	1.001–1.004	< 0.01	1.002	1.001–1.002	< 0.01
eGFR ≥ 90 mL/min/1.73 m^2^				Reference	–	–
60 ≤ eGFR < 90 mL/min/1.73 m^2^				1.160	0.726–1.843	0.541
eGFR < 60 mL/min/1.73 m^2^				2.190	1.118–4.316	0.022

*Abbreviations*: *AKI* acute kidney injury, *ICU* intensive care unit, *eGFR* estimated glomerular filtration rate

**Table 5:** Multivariate cox regression analysis for the risk factors of progressive CKD

	Age < 65 years			Age ≥ 65 years		
	OR	95%CI	*P*-value	OR	95% CI	*P*-value
Age	1.040	1.010–1.064	0.007	1.050	1.008–1.090	0.018
DM	3.310	2.020–5.435	< 0.01	3.510	2.381–5.173	< 0.01
eGFR ≥ 90 mL/min/1.73 m^2^	Reference	–	–	Reference	–	–
60 ≤ eGFR < 90 mL/min/1.73 m^2^	1.690	1.071–2.669	0.024	1.380	0.856–2.209	0.187
eGFR < 60 mL/min/1.73 m^2^	2.890	1.204–6.964	0.018	2.870	1.472–5.580	< 0.01
Postoperative AKI	4.940	3.035–8.042	< 0.01			
ICU stay	1.002	1.001–1.004	< 0.01	1.002	1.001–1.003	< 0.01

*Abbreviations*: *eGFR* estimated glomerular filtration rate, *AKI* acute kidney injury, *ICU* intensive care unit, *CKD* chronic kidney disease

#### Age ≥ 65 years

Multivariate logistic regression analysis showed that age, gender (male), BMI, intraoperative hypotension and LCOS were independent risk factors of AKI after cardiac surgery (Table 3).

Multivariate Cox regression analysis showed that diabetes, length of ICU stay and eGFR < 60 mL/min/1.73 m^2^ were risk factors for 3-year mortality (Table 4) and age, diabetes, eGFR < 60 mL/min/1.73 m^2^ and length of ICU stay were independent risk factors of 3-year progressive CKD (Table 5).

## Discussion

It is commonly accepted that preoperative renal dysfunction is a risk factor for AKI, in-hospital mortality or even long-term outcomes after cardiac surgery [12]. Also “occult renal insufficiency” defined as SCr ≤ 100 μm with CrCl ≤  60 mL/min with an incidence rate of up to 13% has been shown to be associated with RRT [13], which is in accordance with our findings, that for patients with normal SCr under the age of 65 years, preoperative eGFR < 60 mL/min/1.73 m^2^ was an independent risk factor for AKI. However, interestingly for patients over the age of 65 years, preoperative eGFR< 60 ml/min/1.73 m^2^ was not an independent risk factor for AKI in our study, but was still a risk factor for 3-year death and progressive CKD. The senescent kidney already shares morphological features of CKD [7], and the weighing of aging was heavier than eGFR in a multivariate regression analysis, which may explain the result.

However, most studies about preoperative renal dysfunction usually studied patients with eGFR < 60 mL/min /1.73 m^2^ [6, 14], but the risk of patients with mild decreased eGFR (60~90 mL/min /1.73 m^2^) and normal SCr is less evaluated. In the study by Howell et al. including 7621 consecutive patients who underwent CABG, valve surgery or combined procedures, eGFR ≥  90 mL/min /1.73 m^2^ were considered normal renal function (reference group), eGFR 60–90 mL/min/1.73 m^2^ were mild renal dysfunction (group 2), eGFR 30–59 mL/min/ 1.73 m^2^ were moderate renal dysfunction (group 3) and eGFR 15–29 mL/min/1.73 m^2^ were severe renal dysfunction (group 4) and the results showed that eGFR of 60–90 mL/min/1.73 m^2^ (mild renal dysfunction) was an independent predictor of in-hospital and late mortality as well as cardiovascular complications [15], which is in line with our results, which revealed that for patients with normal SCr under the age of 65 years preoperative 60 ≤ eGFR< 90 mL/min/1.73 m^2^ values became a risk factor for 3-year progressive CKD.

The mechanism why mild renal dysfunction contributes to poor outcomes is not clear. A possible explanation is that patients with impaired eGFR may have a more advanced cardiovascular disease and a reduced cardiac output before surgery. Along with aging, inflammatory mediators, endothelial dysfunction, left ventricular hypertrophy, all factors may contribute to the poor outcomes [16] and when eGFR values decreased to 60~90 mL/min/1.73 m^2^, it may be not obvious yet for the kidney to develop AKI, but the kidneys may be less able to recover from the injurious event of surgery.

The cause of preoperative decreased eGFR is complicated and different for eGFR 60~90 mL/min/1.73 m^2^ and eGFR < 60 mL/min/1.73 m^2^ cases. For patients with eGFR 60~90 mL/min/1.73 m^2^, aging and increased BMI may be the main causes for decreased eGFR [7, 17, 18]. In patients over the age of 65 years, the proportion of hidden renal dysfunction was up to 67.0% in our study. It is considered that intimal thickening and decreased compliance of the renal vasculature contribute to observed 10% decreases in renal blood flow (RBF) per decade of life [19] and a decline in eGFR has been observed in patients without histological evidence of nephrosclerosis [20]. Also our results showed that though the rate of abnormalities in urinalysis and renal ultrasound was significantly higher in the eGFR < 60 mL/min/1.73 m^2^ group, there was no statistical difference between 60 ≤ eGFR < 90 mL/min/1.73 m^2^ and no renal dysfunction cases, which confirmed that most patients with eGFR 60–90 mL/min/1.73 m^2^ have no substantial damage. However, 44.0% of our patients < 65 years old had hidden renal dysfunctions. Besides age-dependent changes in kidney structure and function, increased exposure to comorbidities including cardiac-renal syndrome (type II, IV), hypertension, diabetes and nephrotoxic drugs may together increase the susceptibility of the kidney to develop AKI. The main cause may be the hemodynamic instability caused by cardiac-renal syndrome, since in our study, the proportion of New York Heart Association (NYHA) > II of the other three groups were significantly higher than in the no renal dysfunction group. Proportions of hypertension and diabetes also increased along with the decreased eGFR. Thus non-age dependent hidden renal dysfunction may be functional and reversible, and efforts could be made for better prevention of AKI.

Our study has some limitations. First, it was an observational and retrospective study in a single center, possibly not able to identify all potential confounding factors. Furthermore, mild renal dysfunction is always poorly defined. Our study used the cut-off of SCr ≤ 1.2 mg/dL (female)/1.5 mg/dL (male) which was arbitrarily chosen from the normal value of our hospital. We chose it because our aim was to raise the awareness of surgeons to concern about patients with conventionally regarded normal SCr but decreased eGFR,

## Conclusions

Known renal dysfunction (eGFR < 60 mL/min/1.73 m^2^) was a significant risk factor of progressive CKD for both ≥65 and < 65 year old patients. However, hidden renal dysfunction (60 ≤ eGFR < 90 mL/min/1.73 m^2^) correlated with progressive CKD only in < 65 year old patients. Awareness should be raised for these patient group.

## Data Availability

The datasets used and/or analysed during the current study are available from the corresponding author on reasonable request.

## Abbreviations

AKI: Acute kidney injury
BMI: Body mass index
BUN: Blood urea nitrogen
CABG: Coronary artery bypass grafting
CKD: Chronic kidney disease
CPB: Cardiopulmonary bypass
DM: Diabetes mellitus
eCrCl: estimated creatinine clearance
eGFR: estimated glomerular filtration rate
ICU: Intensive care unit
LVEF: Left ventricular ejection fraction
NYHA: New York Heart Association
OPCAB: Off-pump coronary artery bypass
PCr: Plasma creatinine
RBF: Renal blood flow
RRT: Renal replacement therapy
SCr: Serum creatinine
